# Mindfulness and mechanisms of attention in a neutral and palatable food context

**DOI:** 10.3389/fpsyg.2024.1346839

**Published:** 2024-08-02

**Authors:** Zsófia Logemann-Molnár, Anna Veres-Székely, Zsolt Demetrovics, H. N. Alexander Logemann

**Affiliations:** ^1^Doctoral School of Psychology, ELTE, Eötvös Loránd University, Budapest, Hungary; ^2^Institute of Psychology, ELTE, Eötvös Loránd University, Budapest, Hungary; ^3^MTA-ELTE Lendület Adaptation Research Group, Institute of Psychology, ELTE, Eötvös Loránd University, Budapest, Hungary; ^4^Centre of Excellence in Responsible Gaming, University of Gibraltar, Gibraltar, Gibraltar; ^5^College of Education, Psychology and Social Work, Flinders University, Adelaide, SA, Australia; ^6^Department of Clinical, Neuro and Developmental Psychology, Vrije Universiteit Amsterdam, Amsterdam, Netherlands

**Keywords:** mindfulness, attentional bias, attention, reward, palatable food, EEG

## Abstract

**Introduction:**

Mindfulness has been associated with benefits on cognitive processes, including attention. However, the exact relationship between mindfulness, components of attention, and the role of reward context has not yet been fully elucidated, which is relevant, especially in the context of addiction. In the current study, we specifically evaluated the relationship between dispositional mindfulness and the balance between voluntary (top-down), and stimulus-driven (bottom-up) attention. In addition, we explored whether the relationship was mediated by asymmetry of frontal brain activity, an index of approach tendencies, and varies as a function of reward context.

**Methods:**

In total, 95 participants (30 male, 65 female) with a mean age of 25.87 (SD = 7.38) participated. Resting-state electrophysiological activity was recorded using EEG, and participants were assessed on dispositional mindfulness, and performed the visuospatial cueing (VSC) task, which indexed voluntary- and stimulus-driven attention in a neutral and palatable food (reward) context. In the endogenous VSC task, a central cue signals the likely location of a subsequent target. The validity effect represents the benefit of valid cueing relative to the costs of invalid cueing in terms of response time.

**Results and discussion:**

Dispositional mindfulness was associated with a reduced validity effect, plausibly reflecting a combination of reduced voluntary attention and increased stimulus-driven attention, irrespective of condition. The relationship between dispositional mindfulness and visuospatial attention could not be explained by asymmetry of frontal brain activity.

## 1 Introduction

Mindfulness is commonly defined as the capacity, or ability of individuals to be conscious of and attend internal and external events in an open discerning way, without judging these experiences (Shapiro and Carlson, 2009). Mindfulness has been associated with improved cognitive performance, including inhibitory control and attention (Zoogman et al., 2015; Gallant, 2016; Klingbeil et al., 2017; Quaglia et al., 2019; Vekety et al., 2021). However, the moderating role of reward context is not yet fully understood, which is of importance to disorders of addiction. Specifically, addiction is in part characterized by attentional bias for reward-associated stimuli which contributes to associated maladaptive behavior (Robinson et al., 2016; Volkow et al., 2017). However, studies on the effects of mindfulness regarding attentional bias in a reward context are relatively mixed. Some studies suggest that attentional bias is reduced following mindfulness interventions (Garland et al., 2012; Alamout et al., 2020), and other studies suggest enhanced attentional bias for reward-associated stimuli (Delgado-Pastor et al., 2013; Sanger et al., 2018; Isbel et al., 2019; Logemann-Molnár et al., 2022). The aim of the current work was to address this apparent discrepancy in a controlled lab environment.

The neuroanatomical correlates of mindfulness and their association with executive functions are not yet fully understood, but studies suggest that the brain mechanism overlaps between dispositional mindfulness and mindfulness training-induced mindfulness (Bilevicius et al., 2017; Feruglio et al., 2021). Previous studies incorporating brain activity measures have shown that mindfulness is associated with suppressed activity within the Default Mode Network, which is inversely associated with activity in the Salience Network (Bilevicius et al., 2017; Feruglio et al., 2021). This may translate to mindfulness-associated enhanced susceptibility for salient stimuli and subsequent approach tendencies (Bilevicius et al., 2017; Feruglio et al., 2021). This effect is further supported by studies that show that mindfulness is associated with more left relative to right frontal brain activity, indexed by frontal alpha asymmetry using electroencephalography (EEG) (Keune et al., 2013; Moynihan et al., 2013), which has also been associated with enhanced approach tendencies (Kelley et al., 2017). However, it should be noted that other studies did not find such effect (Keune et al., 2011; Szumska et al., 2021), and the effect may be state dependent (Keune et al., 2013).

Based on the above, mindfulness may be associated with enhanced attentional bias for salient stimuli. Behavioral studies support this notion. Studies that implemented an oddball paradigm show mindfulness-associated attentional capture of deviant infrequent, but salient stimuli in the context of repetitive stimuli (Delgado-Pastor et al., 2013; Sanger et al., 2018; Isbel et al., 2019). ^132425^ However, studies on addiction that employed variants of a visual probe task and have focused on the association between mindfulness and attentional bias for reward related stimuli, may seem incongruent at first glance. Typically, in these studies, mindfulness reduced attentional bias for reward-associated stimuli (Garland et al., 2012; Alamout et al., 2020). One plausible explanation for this apparent discrepancy may be related to differences in the expectedness of the salient, reward-related stimulus. It should be noted that visuospatial attention consists of two main components, voluntary (top-down) attentional orienting, and stimulus-driven (bottom-up) reflexive attentional (re-)orienting (Corbetta and Shulman, 2002; Corbetta et al., 2008). Voluntary attention allows for the conscious focus of attention to potentially relevant stimuli in our environment, whereas stimulus-driven attentional (re-)orienting allows for attentional disengagement and subsequent shift towards unanticipated, yet relevant, salient stimuli (Corbetta and Shulman, 2002; Corbetta et al., 2008). It seems plausible that mindfulness exerts differential effects on these components. To elaborate, reward-associated stimuli in oddball paradigms are commonly infrequently presented and relatively unexpected. In such case, results seem to reflect that mindfulness is associated with enhanced stimulus-driven attentional capture of such stimuli. In contrast, reward-associated stimuli in visual probe paradigms are expected and frequently presented. In such studies, mindfulness is associated with reduced voluntary attention towards such stimuli.

One excellent paradigm to investigate and differentiate voluntary attention from stimulus-driven attention to unexpected relevant stimuli is the Posner paradigm, also called the visuospatial cueing task (Posner et al., 1980). In such task, a cue indicates the likely location of a subsequent target to which a response is required. In a minority of trials, the target is presented at a location opposite as indicated by the cue. Generally, individuals respond faster to targets that are validly cued, and thus presented in the attended visual hemifield as opposed to targets that are invalidly cued and presented in the unattended visual hemifield. The relevant index is the validity effect, which is operationalized as the difference in response time to validly relative to invalidly cued targets (Posner et al., 1980). The validity effect represents the benefit in terms of speeded response times due to valid cueing relative to response time costs in the context of invalid cueing. It can be argued that enhanced voluntary attention in response to valid cueing results in faster responses and an enhanced validity effect. In contrast, stronger stimulus-driven attention is associated with enhanced attentional disengagement and reorienting in the context of invalid cueing, and results in a reduction of the validity effect (Corbetta and Shulman, 2002; Corbetta et al., 2008). In the VSC task, reward context can be operationalized by target characteristics (Failing and Theeuwes, 2014; Tsegaye et al., 2022). Specifically, an intrinsic reward context can be operationalized by target stimuli representing palatable food, which is known to trigger reward circuity in the brain, similarly to monetary target stimuli (Yousuf et al., 2018). Indeed, it may be questioned whether this will also translate into observable effects in normal healthy samples. In that vein, it should be noted that a recent systematic review showed no consistent difference in attentional bias comparing overweight/obese individuals to healthy controls (Hagan et al., 2020). This is also supported by our previous online study that suggested enhanced attentional bias in a palatable food relative to a neutral context, irrespective of BMI (Tsegaye et al., 2020).

Integrating the results from the aforementioned studies, the following hypotheses were postulated. Firstly, it was hypothesized that mindfulness would be associated with a reduced validity effect, reflecting a combination of reduced voluntary attention and increased stimulus-driven attention. Secondly, it was expected that this effect would be higher in a reward context, operationalized by palatable food associated stimuli, relative to the neutral context. Lastly, we explored the relationship between mindfulness and frontal alpha asymmetry as a brain activity index of approach tendency.

## 2 Methods

### 2.1 Participants

The sample size was determined using G*power (Faul et al., 2007, 2009). Detecting the relevant within/between-subjects interactions of small to medium effect size (Cohen’s *f* = 0.15) with power and alpha set at 0.80, and 0.05 respectively, required a minimal total sample size of 75. To account for potential attrition, and exclusion of cases due to erroneous data, we recruited a higher number of participants. Participants were recruited via social media, and from the student population. Participants were offered either a modest monetary incentive or course credit. Specifically, in total 95 participants (30 male, 65 female) with a mean age of 25.87 (SD = 7.38) participated. Individuals under 18 years old, and those diagnosed with a neurological and/or psychological disorder could not participate. All participants were fully informed and provided their written informed consent prior to any procedures. The study was approved by the Research Ethics Committee of the of the Institute of Psychology, Eötvös Loránd University (ELTE), and was conducted in accordance with the Declaration of Helsinki and its later amendments.

### 2.2 Materials

#### 2.2.1 Platform

Demographic questions, and the Mindful Attention Awareness Scale (MAAS) self-report scale were implemented in Psytoolkit (Stoet, 2010; Stoet, 2017). The visuospatial cueing task was implemented in Opensesame (Mathôt et al., 2012).

#### 2.2.2 Mindful attention awareness scale

The MAAS self-report questionnaire consists of 15 items and yields a measure of dispositional mindfulness (Brown and Ryan, 2003). The outcome variable is computed as the average of the item scores. A high scale score is thought to represent a relative higher level of dispositional mindfulness. Reliability is good, with a Cronbach’s alpha for the scale exceeding 0.80 (Brown and Ryan, 2003; van Dam et al., 2011).

#### 2.2.3 Visuospatial cueing task

**Figure 1:**
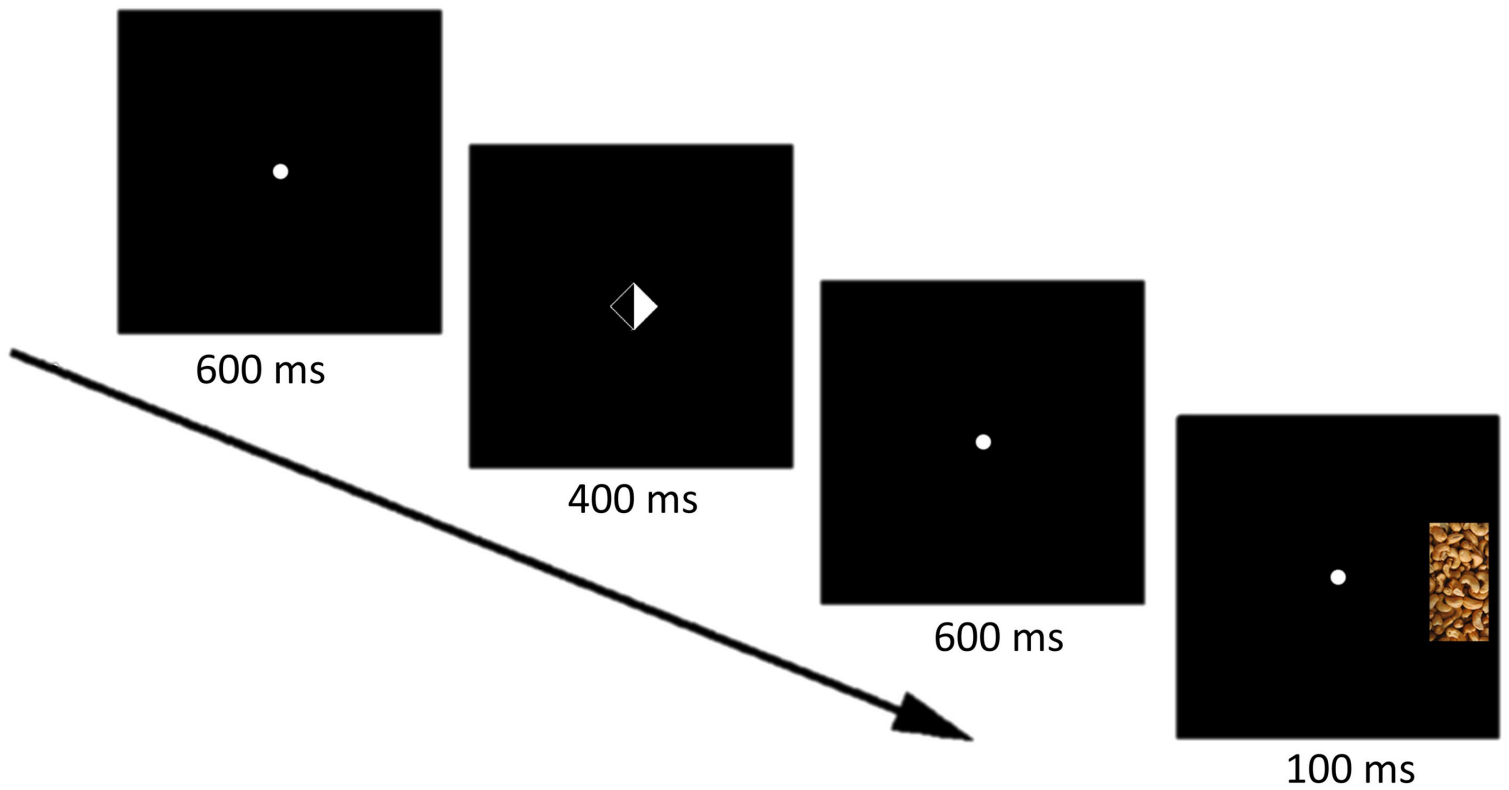
Valid trial in the food condition.

The visuospatial cueing task (VSC) was an adaptation of the original conceptualization by Posner et al. (1980). Figure 1 shows a typical trial. In the VSC task, the diamond shaped cue signals the likely location of the subsequent target. The distance from participants’ eyes to the screen was approximately 65 cm. The cue could point to the left, to the right, or could be neutral (empty, not informative). Subsequent to the cue, the target was presented in the left or right visual hemifield. Participants were required to indicate via button press (“A,” or “L”) whether the target was long (100×200 pixels) or short (100×120 pixels). Total trial duration was 3,100 ms. Three types of trials were included. In a valid trial, the target is presented at the visual hemifield indicated by the cue. In a neutral trial, the cue is not informative with respect to the location of the target. Lastly, in an invalid trial, the target is presented at the opposite visual hemifield. The experiment included one practice block, and two experimental blocks per condition (neutral or food). The practice block included nine valid trials, three neutral trials, and two invalid trials. Each experimental block consisted of 160 trials, consisting of 32 invalid trials, 32 neutral trials, and 96 valid trials. The only difference between the conditions was the type of target. In the neutral condition, targets were gray bars. In the food context, the targets consisted of depictions either chips, chocolate-chip cookies, nuts or chocolate. The order of conditions and response-target assignment was randomized across participants to control for non-specific (e.g., training/fatigue) effects. The relevant outcome variables are the average response time to validly, neutral, and invalidly cued targets in the neutral and food context. The validity effect represents the benefit of cueing on response time, specifically the reduced response time to validly cued targets relative to invalidly cued targets.

#### 2.2.4 EEG data acquisition and preprocessing

The Mind Media Nexus 32 amplifier was used together with a 10/20, 21-channel (Ag/AgCl electrodes) cap. Electrophysiological activity was recorded by using the Mind Media NeXus-32 amplifier in combination with a 21-channel cap with Ag/AgCl electrodes (Mind Media B.V., The Netherlands). Data acquisition was at 512 samples per second, using the common average. Eye movements were recorded at the outer canthi of both eyes (HEOG), and supra-and infraorbital to the eye (VEOG). FAA was computed as follows, in accordance with Smith et al. (2017): specifically, raw EDF+ format data was filtered with a high pass filter of 0.5 Hz, and 40 Hz low pass filter with a notch filter of 50 Hz. The first and last 10 s of EEG data were discarded to prevent potential artifacts. Data was segmented to two-second epochs and corrected for eye-movement artifacts using Independent Component Analysis (ICA). Subsequently, the epochs were whole segment baseline corrected and epochs that included remaining artifacts, defined as +/− 75 microvolt relative to baseline were rejected. Power Spectral Density (PSD) was computed using Fast Fourier Transform (FFT) with a 10% Hanning window. These epochs were averaged, and mean activity (power) in the alpha frequency band (8–13 Hz) was computed for the contralateral F3 and F4 channels. The rank ordered distribution of alpha power is generally skewed, and to meet assumptions of the parametric tests, we corrected for skew using natural log transform (Smith et al., 2017). Finally, the FAA outcome variable was calculated by subtracting log transformed alpha at F3 from F4.

### 2.3 Procedures

Upon arrival at the lab, participants signed the informed consent form, and were seated in a comfortable chair in a dimly lit and sound attenuated room. The EEG cap was placed, and conductance gel was applied between the electrodes and scalp. After the signal was checked, 5 min eyes open and 5 min eyes closed resting state EEG was recorded. Subsequently, participants filled out the questionnaires to assess the standard demographic variables and MAAS score. Afterwards, participants performed the VSC task and Stop Signal Task (associated data was not part of the current report but will be presented elsewhere). Participants were provided with short breaks between the blocks, and tasks.

### 2.4 Statistical analyses

Data processing was performed using R (R Core Team, 2018), and statistical analyses were performed with SPSS (IBM Corp, 2019). Repeated measures ANCOVAs were planned *a priori*, with MAAS score as between-subjects factor, and condition (levels: neutral/reward), and validity (levels: valid/neutral/invalid) as within-subjects factors. We performed two tests of correlation (Pearson *r*) to test the relationship between MAAS score and FAA, for the eyes-open and eyes-closed condition. Alpha was set at 0.05. In case the assumption of sphericity was violated, Greenhouse–Geisser correction was applied.

## 3 Results

**Figure 2:**
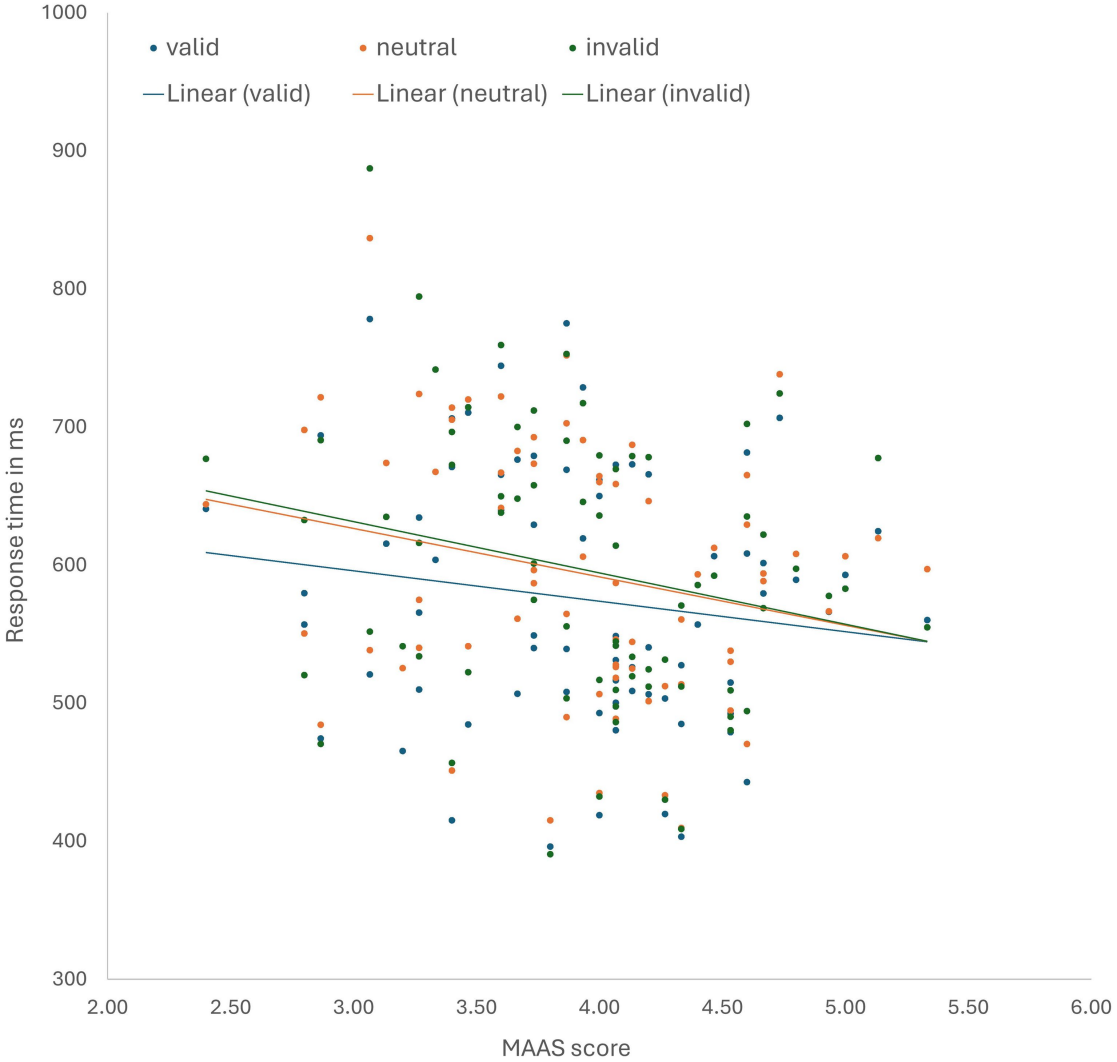
Response time in ms as a function of validity and MAAS score, collapsed over condition.

For the main VSC task analyses, participants (N = 72) were included that completed the VSC task and were engaged with the task, indicated by performance above chance level during all conditions and trial-types (proportion correct >0.5). We should note that the main results of the selected sample did not differ in terms of significance from the explorative whole group analyses. The MAAS score ranged from 2.40 to 5.33 (*M* = 3.93, SD = 0.60). Dispositional mindfulness did not affect the relationship between reward context (neutral/food) and validity (valid/neutral/invalid) regarding response time, *F*(1.79,125.41) = 0.30, *p* = 0.715, η_p_^2^ = 0.004. Hence, this does not support that the relationship between dispositional mindfulness and voluntary relative to stimulus-driven driven attention differs between the conditions. However, as evident in Figure 2, dispositional mindfulness significantly reduced voluntary attention relative to stimulus-driven attention irrespective of reward context, as indicated by a significant interaction between dispositional mindfulness and validity (valid/neutral/invalid) regarding response time, *F*(1.77,124.02) = 3.73, *p* = 0.032, η_p_^2^ = 0.051. This was also confirmed by subsequent post-hoc analysis, which revealed a reduced response time difference between validly cued targets and invalidly cued targets as a function of dispositional mindfulness level, *F*(1,70) = 4.70, *p* = 0.034, η_p_^2^ = 0.063. There was an overall effect of cuing on response time. Specifically, the main effect of validity (valid/neutral/invalid) regarding response time was significant, *F*(1.77,124.02) = 6.75, *p* = 0.002, η_p_^2^ = 0.088. Post-hoc testing revealed that responses were significantly faster for validly cued targets relative to invalidly cued targets [*F*(1,70) = 8.51, *p* = 0.005, η_p_^2^ = 0.108], underscoring the validity of the paradigm. There was no main effect of dispositional mindfulness regarding response time, *F*(1, 70) = 3.04, *p* = 0.086, η_p_^2^ = 0.042. Lastly, There was no significant association between dispositional mindfulness and frontal alpha asymmetry in both the eyes closed and eyes open condition, respectively r(95) = 0.034, *p* = 0.741, and r(95) = −0.084, *p* = 0.417. The significance did not change after the exclusion of outliers (defined as exceeding 2 SD from the mean).

## 4 Discussion

The current study focused on the relationship between dispositional mindfulness and voluntary and stimulus driven attention in contexts that differed in terms of intrinsic reward. The results partly confirmed our hypotheses. Specifically, dispositional mindfulness was associated with a reduction of the validity effect, plausibly reflecting a combination of reduced voluntary attention and enhanced stimulus-driven attention. In contrast to our hypotheses, this effect did not differ as a function of reward context. In addition, the observed relationship between dispositional mindfulness and visuospatial attention could not be explained by asymmetry of frontal brain activity.

Results of the current study suggest that mindfulness differentially impacts voluntary and stimulus-driven attention as indicated by the reduced validity effect. Interestingly, we did not find that reward context moderated the effect of dispositional mindfulness on the validity effect. It might be argued that such effect might be restricted to individuals affected by addiction and obesity (noting the considerable overlap of the latter with addiction in terms of behavioural challenges as well as the associated brain mechanism; Tsegaye et al., 2020). On the other hand, palatable food stimuli (intrinsically rewarding stimuli), have been shown to induce attentional bias in healthy participants, not only in obesity (Hagan et al., 2020). Either way, our sample consisted of healthy participants, and the paradigm included a narrow set of palatable food stimuli. Hence, we cannot fully exclude the possibility that effects might be different in addiction using a different set of stimuli associated with the drug of choice.

We also explored the potential relationship between dispositional mindfulness and asymmetry of frontal brain activity, indexed by FAA. If such relationship would be evident, FAA could be an interesting target for neuromodulatory approaches such as Transcranial Alternating Current Stimulation, Unilateral Muscle Contraction, or perhaps EEG-feedback, to potentially supplement mindfulness training (Kelley et al., 2017). However, results did not show evidence of such relationship. Though this could indicate that the relationship between dispositional mindfulness and visuospatial attention cannot be explained by asymmetry of frontal brain activity, appropriate nuance should be applied. It should be underscored that we did not perform a mindfulness intervention but assessed dispositional mindfulness and did not select individuals based on high vs. low mindfulness, which could be considered a limitation. As such, the range of dispositional mindfulness levels might have been too limited to detect an effect on FAA. On the other hand, it remains a possibility that the relationship between dispositional mindfulness and visuospatial attention is best explained by a different electrophysiological mechanism.

Some other limitations of the current study should also be noted. Based on previous studies that suggested a moderating role of reward context regarding the relationship between subject characteristics and executive control (Houben et al., 2014; Tsegaye et al., 2020), we included depictions of both savory and sweet snacks as target stimuli for the food context. However, we did not control for individual differences in food preference, which may result in enhanced variability and associated reduced statistical power to detect a potential moderating effect. Also, we must be careful with generalizing across other reward contexts. As argued before, effects may vary as a function of the exact operationalization of reward context.

Lastly, it may be argued that our implementation of the reward context does not only differ in terms of reward from the neutral context, but also in terms of stimulus complexity. It has been shown that higher stimulus complexity results in increased response times, due to enhanced processing that is required for such stimuli (Gajewski and Falkenstein, 2013). However, our data does not show a significant effect of reward context on mean response time (collapsed across trial-type), *F*(1,70) = 2.20, *p* = 0.143, η_p_^2^ = 0.030.

In conclusion, together with previous observations, our results imply that mindfulness reduces voluntary goal-directed (top-down) attention while enhancing stimulus-driven (bottom-up) attention to salient unexpected stimuli in our environment.

## Data availability statement

The datasets presented in this study can be found in online repositories. The names of the repository/repositories and accession number(s) can be found below: Open Science Framework public repository: DOI 10.17605/OSF.IO/4965P.

## Ethics statement

The studies involving humans were approved by Research Ethics Committee of the Institute of Psychology, Eötvös Loránd University (ELTE). The studies were conducted in accordance with the local legislation and institutional requirements. The participants provided their written informed consent to participate in this study.

## Author contributions

ZL-M: Conceptualization, Formal analysis, Investigation, Methodology, Project administration, Visualization, Writing – original draft, Writing – review & editing. AV-S: Conceptualization, Funding acquisition, Methodology, Writing – review & editing. ZD: Conceptualization, Funding acquisition, Methodology, Resources, Writing – review & editing. HL: Conceptualization, Formal analysis, Funding acquisition, Methodology, Project administration, Resources, Software, Visualization, Writing – review & editing.

## References

[ref1] Alamout M. M. Rahmanian M. Aghamohammadi V. Mohammadi E. Nasiri K. (2020). Effectiveness of mindfulness based cognitive therapy on weight loss, improvement of hypertension and attentional bias to eating cues in overweight people. Int. J. Nurs. Sci. 7, 35–40. doi: 10.1016/j.ijnss.2019.12.010, PMID: 32099857 PMC7031128

[ref2] Bilevicius E. Smith S. D. Kornelsen J. (2017). Resting-state network functional connectivity patterns associated with the mindful attention awareness scale. Brain Connect. 8, 40–48. doi: 10.1089/brain.2017.0520 29130326

[ref3] Brown K. W. Ryan R. M. (2003). The benefits of being present: mindfulness and its role in psychological well-being. J. Pers. Soc. Psychol. 84, 822–848. doi: 10.1037/0022-3514.84.4.822 12703651

[ref4] Corbetta M. Patel G. Shulman G. L. (2008). The reorienting system of the human brain: from environment to theory of mind. Neuron 58, 306–324. doi: 10.1016/j.neuron.2008.04.017, PMID: 18466742 PMC2441869

[ref5] Corbetta M. Shulman G. L. (2002). Control of goal-directed and stimulus-driven attention in the brain. Nat. Rev. Neurosci. 3, 201–215. doi: 10.1038/nrn755 11994752

[ref6] Delgado-Pastor L. C. Perakakis P. Subramanya P. Telles S. Vila J. (2013). Mindfulness (Vipassana) meditation: effects on P3b event-related potential and heart rate variability. Int. J. Psychophysiol. 90, 207–214. doi: 10.1016/j.ijpsycho.2013.07.006, PMID: 23892096

[ref7] Failing M. F. Theeuwes J. (2014). Exogenous visual orienting by reward. J. Vis. 14:6. doi: 10.1167/14.5.6, PMID: 24819737

[ref8] Faul F. Erdfelder E. Buchner A. Lang A. G. (2009). Statistical power analyses using G*power 3.1: tests for correlation and regression analyses. Behav. Res. Methods 41, 1149–1160. doi: 10.3758/BRM.41.4.1149, PMID: 19897823

[ref9] Faul F. Erdfelder E. Lang A. G. Buchner A. (2007). G*power 3: a flexible statistical power analysis program for the social, behavioral, and biomedical sciences. Behav. Res. Methods 39, 175–191. doi: 10.3758/BF03193146, PMID: 17695343

[ref10] Feruglio S. Matiz A. Pagnoni G. Fabbro F. Crescentini C. (2021). The impact of mindfulness meditation on the wandering mind: a systematic review. Neurosci. Biobehav. Rev. 131, 313–330. doi: 10.1016/j.neubiorev.2021.09.032, PMID: 34560133

[ref11] Gajewski P. D. Falkenstein M. (2013). Effects of task complexity on ERP components in go/Nogo tasks. Int. J. Psychophysiol. 87, 273–278. doi: 10.1016/j.ijpsycho.2012.08.007 22906814

[ref12] Gallant S. N. (2016). Mindfulness meditation practice and executive functioning: breaking down the benefit. Conscious. Cogn. 40, 116–130. doi: 10.1016/j.concog.2016.01.005, PMID: 26784917

[ref13] Garland E. L. Boettiger C. A. Gaylord S. Chanon V. W. Howard M. O. (2012). Mindfulness is inversely associated with alcohol attentional Bias among recovering alcohol-dependent adults. Cognit Ther Res 36, 441–450. doi: 10.1007/s10608-011-9378-7, PMID: 23280000 PMC3532517

[ref14] Hagan K. E. Alasmar A. Exum A. Chinn B. Forbush K. T. (2020). A systematic review and meta-analysis of attentional bias toward food in individuals with overweight and obesity. Appetite 151:104710. doi: 10.1016/j.appet.2020.104710, PMID: 32298701

[ref15] Houben K. Nederkoorn C. Jansen A. (2014). Eating on impulse: the relation between overweight and food-specific inhibitory control. Obesity (Silver Spring) 22, E6–E8. doi: 10.1002/oby.20670, PMID: 24910860

[ref16] IBM Corp. IBM SPSS statistics for windows, version 26.0. (2019)

[ref17] Isbel B. D. Lagopoulos J. Hermens D. F. Summers M. J. (2019). Mental training affects electrophysiological markers of attention resource allocation in healthy older adults. Neurosci. Lett. 698, 186–191. doi: 10.1016/j.neulet.2019.01.029, PMID: 30659914

[ref18] Kelley N. J. Hortensius R. Schutter D. J. L. G. Harmon-Jones E. (2017). The relationship of approach/avoidance motivation and asymmetric frontal cortical activity: a review of studies manipulating frontal asymmetry. Int. J. Psychophysiol. 119, 19–30. doi: 10.1016/j.ijpsycho.2017.03.001, PMID: 28288803

[ref19] Keune P. M. Bostanov V. Hautzinger M. Kotchoubey B. (2011). Mindfulness-based cognitive therapy (MBCT), cognitive style, and the temporal dynamics of frontal EEG alpha asymmetry in recurrently depressed patients. Biol. Psychol. 88, 243–252. doi: 10.1016/j.biopsycho.2011.08.008 21884751

[ref20] Keune P. M. Bostanov V. Hautzinger M. Kotchoubey B. (2013). Approaching dysphoric mood: state-effects of mindfulness meditation on frontal brain asymmetry. Biol. Psychol. 93, 105–113. doi: 10.1016/j.biopsycho.2013.01.016, PMID: 23410762

[ref21] Klingbeil D. A. Renshaw T. L. Willenbrink J. B. Copek R. A. Chan K. T. Haddock A.. (2017). Mindfulness-based interventions with youth: a comprehensive meta-analysis of group-design studies. J. Sch. Psychol. 63, 77–103. doi: 10.1016/j.jsp.2017.03.006, PMID: 28633940

[ref22] Logemann-Molnár Z. Veres-Székely A. Demetrovics Z. Logemann H. N. A. (2022). Seeing attractive faces challenges inhibitory control, especially when mindful. PLoS One 17:e0273913. doi: 10.1371/journal.pone.0273913, PMID: 36048784 PMC9436117

[ref23] Mathôt S. Schreij D. Theeuwes J. (2012). OpenSesame: an open-source, graphical experiment builder for the social sciences. Behav. Res. Methodst 44, 314–324. doi: 10.3758/s13428-011-0168-7, PMID: 22083660 PMC3356517

[ref24] Moynihan J. A. Chapman B. P. Klorman R. Krasner M. S. Duberstein P. R. Brown K. W.. (2013). Mindfulness-based stress reduction for older adults: effects on executive function, frontal alpha asymmetry and immune function. Neuropsychobiology 68, 34–43. doi: 10.1159/000350949, PMID: 23774986 PMC3831656

[ref25] Posner M. I. Snyder C. R. Davidson B. J. (1980). Attention and the detection of signals. J. Exp. Psychol. 109, 160–174. doi: 10.1037/0096-3445.109.2.160 7381367

[ref26] Quaglia J. T. Zeidan F. Grossenbacher P. G. Freeman S. P. Braun S. E. Martelli A.. (2019). Brief mindfulness training enhances cognitive control in socioemotional contexts: behavioral and neural evidence. PLoS One 14:e0219862. doi: 10.1371/journal.pone.0219862, PMID: 31323050 PMC6641506

[ref27] R Core Team (2018). R: A language and environment for statistical computing. Vienna, Austria: R Foundation for Statistical Computing.

[ref28] Robinson M. J. F. Fischer A. M. Ahuja A. Lesser E. N. Maniates H. (2016). Roles of ‘wanting’ and ‘liking’ in motivating behavior: gambling, food, and drug addictions. Curr. Top. Behav. Neurosci. 27, 105–136. doi: 10.1007/7854_2015_387, PMID: 26407959

[ref29] Sanger K. L. Thierry G. Dorjee D. (2018). Effects of school-based mindfulness training on emotion processing and well-being in adolescents: evidence from event-related potentials. Dev. Sci. 21, –e12646. doi: 10.1111/desc.12646, PMID: 29356254 PMC6175003

[ref30] Shapiro S. L. Carlson L. E. (2009). Mindfulness-based psychotherapy. The art and science of mindfulness: Integrating mindfulness into psychology and the helping professions, 45–60. Available at: https://www.apa.org/pubs/books/4317196?tab=4

[ref31] Smith E. E. Reznik S. J. Stewart J. L. Allen J. J. B. (2017). Assessing and conceptualizing frontal EEG asymmetry: an updated primer on recording, processing, analyzing, and interpreting frontal alpha asymmetry. Int. J. Psychophysiol. 111, 98–114. doi: 10.1016/j.ijpsycho.2016.11.005, PMID: 27865882 PMC6449497

[ref32] Stoet G. (2010). PsyToolkit: a software package for programming psychological experiments using Linux. Behav. Res. Methods 42, 1096–1104. doi: 10.3758/BRM.42.4.1096 21139177

[ref33] Stoet G. (2017). PsyToolkit: a novel web-based method for running online questionnaires and reaction-time experiments. Teach. Psychol. 44, 24–31. doi: 10.1177/0098628316677643

[ref34] Szumska I. Gola M. Rusanowska M. Krajewska M. Żygierewicz J. Krejtz I.. (2021). Mindfulness-based cognitive therapy reduces clinical symptoms, but do not change frontal alpha asymmetry in people with major depression disorder. Int. J. Neurosci. 131, 453–461. doi: 10.1080/00207454.2020.1748621 32223344

[ref35] Tsegaye A. Bjørne J. Winther A. Kökönyei G. Cserjési R. Logemann H. N. A. (2020). Attentional bias and disengagement as a function of body mass index in conditions that differ in anticipated reward. J. Behav. Addict. 9, 818–825. doi: 10.1556/2006.2020.00073 33006956 PMC8943657

[ref36] Tsegaye A. Guo C. Stoet G. Cserjési R. Kökönyei G. Logemann H. N. A. (2022). The relationship between reward context and inhibitory control, does it depend on BMI, maladaptive eating, and negative affect? BMC Psychol 10, 1–9. doi: 10.1186/s40359-021-00712-5 34983661 PMC8729126

[ref37] Tsegaye A. Kökönyei G. Baldacchino A. Urbán R. Demetrovic Z. Logemann H. N. A. (2020). The psychological basis of obesity. Obesity and obstetrics. 37–44. doi: 10.1016/B978-0-12-817921-5.00004-7

[ref38] van Dam N. T. Sheppard S. C. Forsyth J. P. Earleywine M. (2011). Self-compassion is a better predictor than mindfulness of symptom severity and quality of life in mixed anxiety and depression. J. Anxiety Disord. 25, 123–130. doi: 10.1016/j.janxdis.2010.08.011 20832990

[ref39] Vekety B. Logemann H. N. A. Takacs Z. K. (2021). The effect of mindfulness-based interventions on inattentive and hyperactive–impulsive behavior in childhood: a meta-analysis. Int. J. Behav. Dev. 45, 133–145. doi: 10.1177/0165025420958192

[ref40] Volkow N. Wise R. A. Baler R. (2017). The dopamine motive system: implications for drug and food addiction. Nat. Rev. Neurosci. 18, 741–752. doi: 10.1038/nrn.2017.130, PMID: 29142296

[ref41] Yousuf M. Heldmann M. Göttlich M. Münte T. F. Doñamayor N. (2018). Neural processing of food and monetary rewards is modulated by metabolic state. Brain Imaging Behav. 12, 1379–1392. doi: 10.1007/s11682-017-9811-y, PMID: 29243121

[ref42] Zoogman S. Goldberg S. B. Hoyt W. T. Miller L. (2015). Mindfulness interventions with youth: a meta-analysis. Mindfulness 6, 290–302. doi: 10.1007/s12671-013-0260-4

